# Oral lichenoid lesion in association with chemotherapy treatment for non-Hodgkin lymphoma or lichen planus? Review of the literature and report of two challenging cases

**DOI:** 10.1186/s13005-022-00333-2

**Published:** 2022-09-06

**Authors:** Letícia Côgo Marques, Laiza Angela de Medeiros Nunes da Silva, Pâmella de Pinho Montovani Santos, Amanda de Almeida Lima Borba Lopes, Karin Soares Cunha, Adrianna Milagres, Rafaela Elvira Rozza-de-Menezes, Arley Silva Junior, Danielle Castex Conde

**Affiliations:** 1 Postgraduate Program in Pathology, School of Medicine, Universidade Federal Fluminense (UFF), Niterói, RJ Brazil; 2 Department of Pathology, School of Medicine, Universidade Federal Fluminense (UFF), Niterói, RJ Brazil

**Keywords:** Lichenoid eruptions, Lichen planus, Mouth disease, Lymphoma, Drug therapy, Antineoplastic agents

## Abstract

**Background:**

The diagnosis of oral lichenoid lesions (OLL) remains a challenge for clinicians and pathologists. Although, in many cases, OLL cannot be clinically and histopathologically distinguishable from oral lichen planus (OLP), one important difference between these lesions is that OLL has an identifiable etiological factor, e.g. medication, restorative material, and food allergy. The list of drugs that can cause OLL is extensive and includes anti-inflammatory drugs, anticonvulsants, antihypertensives, antivirals, antibiotics, chemotherapeutics, among others. This work aimed to perform a literature review of OLL related to chemotherapy drugs and to report two cases of possible OLL in patients with B-cell and T-cell non-Hodgkin lymphomas in use of chemotherapy and adjuvant medications. We also discuss the challenge to clinically and histopathologically differentiate OLL and OLP.

**Case presentation:**

In both cases, oral lesions presented reticular, atrophic, erosive/ulcerated, and plaque patterns. The diagnosis of OLL was initially established in both cases by the association of histopathology and history of onset of lesions after the use of medications. Although the patients have presented a significant improvement in the oral clinical picture for more than 2 years of follow-up, they still have some lesions.

**Conclusion:**

A well-detailed anamnesis associated with the drug history, temporal relationship of the appearance of the lesions, and follow-up of patients are fundamental for the diagnosis of OLL related to drugs. Nevertheless, its differentiation from OLP is still a challenge.

Oral lichen planus (OLP) and oral lichenoid lesion (OLL) represent a heterogeneous group of inflammatory diseases characterized by similar clinical manifestations and histopathological features [1]. Terminologies, classifications, and diagnostic criteria have been discussed and many nomenclatures for these lesions have already been used, which contributed to the difficulty to differentiate these two lesions, and consequently to choose the appropriate therapeutic approach [2].

This work aimed to perform a literature review of OLL related to chemotherapy drugs and to report two cases of possible OLL in patients with B-cell and T-cell non-Hodgkin lymphomas in use of chemotherapy and adjuvant medications. One patient developed lesions after the use of R-CVP chemotherapy protocol and adjuvant medications. The other developed oral lesions after the use of Hyper-CVAD and POMP chemotherapy protocol, and adjuvant medications. We also discuss the challenge to clinically and histopathologically differentiate OLL and OLP.

## Review of the literature

OLP is a mucocutaneous disorder of unknown established etiology that often affects the oral mucosa [3]. It commonly occurs in individuals between 30 and 80 years of age, with an average age of 55.2 years [4], being more prevalent in white women [4–7]. OLL is a chronic inflammatory disease that most often affects women in a wide age range [8–10]. Unlike OLP, the etiology of OLL is generally associated with some identifiable etiological factor, e.g. dental restorative materials, food allergies and the use of medications [11]. The pathogenesis of OLL related to drugs occurs when the drug acts as a hapten, binding to keratinocytes/melanocytes and producing a cytotoxic T lymphocyte response [12].

OLL related to drugs is believed to be uncommon compared to lichenoid skin lesions. However, they are likely to be underreported [1], since the maintenance of drug and the appearance of lesions can vary from days to months [2, 13]. Therefore, it is recommended to investigate the use of medications in the past 12 to 14 months [14]. The list of medications that can cause OLL is extensive and includes non-steroidal anti-inflammatory drugs, anticonvulsants, antihypertensives, antivirals (e.g acyclovir) [1, 2, 15–17], antibiotics (e.g. sulfamethoxazole) [18], chemotherapy agents [19], among others.

Some medications for the treatment of Non-Hodgkin lymphoma (NHL), which is a heterogeneous group of lymphoid malignancies, have been associated with the development of oral and cutaneous lichenoid reactions [14, 20]. After a literature review, we did not find reports associating the use of R-CVP, Hyper-CVAD, and POMP chemotherapy protocols with the appearance of lichenoid lesions. Nevertheless, there are reports of lichenoid lesions in association with protocols that use one of the components of R-CVP [14, 20, 21] and Hyper-CVAD [22, 23], as well as with the use of sulphamethoxazole (prophylactic treatment drugs for infection during chemotherapy) [18].

R-CVP and Hyper-CVAD are protocols widely used in the treatment of NHL [24, 25]. R-CVP consists of the use of the drugs rituximab, cyclophosphamide, vincristine, and prednisone [25]. Hyper-CVAD consists of hyperfractionation of drugs in two courses: odd courses, which include ondansetron, cyclophosphamide, vincristine, doxorubicin, dexamethasone, and mesna; and even courses, composed of methotrexate, folinic acid, methylprednisolone, and cytarabine [24]. The POMP is a chemotherapy maintenance protocol in which the drugs methotrexate, vincristine, and mercaptopurine are used [26].

Rituximab, a chimeric monoclonal antibody that targets the CD20 antigen on B cells, is a medication widely used to treat lymphomas, as it is well tolerated by most patients [20]. Although studies indicate efficacy in the treatment of T cell-mediated inflammatory processes, due to its immunomodulating action on the functions of lymphocytes and their cytokines [12, 27, 28], its use has also been associated with side effects, as well as the onset of OLL [14, 20].

Table 1 shows the reports in the literature of OLL cases in association with the same drugs used by the two patients reported in the present study.

**Table 1:** Reports in the literature of oral lichenoid lesions in association with the same drugs used by the two patients reported in the present study

**Author**	**Age/Sex**	**Drug**	**Time for the appearance of lesions**	**Oral manifestations/ anatomical location**	**Extraoral lesions**	**Management of oral lesions**	**Time for healing**
Kuten-Shorrer [6]	43/F	Rituximab (750mg/m²)	Three months after the 4th dose	Ulcerated and reticular lesions ranging from 0.5 to 4.0 cm, distributed bilaterally and symmetrically on the buccal mucosa, upper labial mucosa, and dorsal and ventral of the tongue	No	Topical dexamethasone solution (5 mg per 5 mL), twice daily, and 40 mg prednisone for 7 days	Complete resolution of the lesions 9-month after the treatment
Giudice et al. [5]	40/F	Rituximab (375 mg)	After the 5th dose	Ulcerated and reticular lesions on buccal mucosa (bilateral and symmetrical) and on the right border of the tongue	Skin	Intralesional injections of 0.5 mL triamcinolone acetonide (Kenacort 40 mg/mL) and systemic corticosteroid	At 6-month follow-up, 9 months after rituximab withdrawal, oral symptomatology did not relapse; skin lesions and joint pain had autonomously healed while oral ulcers did not completely disappear
Kusano et al. [29]	65/M	Bendamustine and rituximab	After the 4th week of treatment	Bullous lesions on lip and oral cavity	Skin, eye, genital and nasal mucosa	Prednisone 5 mg daily	Oral mucosal lesions were intractable after 4-months of treatment
Bronny and Thies [17]	59/M	Sulfamethoxazole	Information not available	Ulcerated lesions (1 cm x 5 mm) near the right lip commissure and left buccal mucosa distal to the commissure. White striae and papules radiated from these ulcers	No	A 1- week course of systemic methylprednisolone decreasing-dosage therapy and viscous lidocaine for symptomatic relief	After one week of treatment, oral lesions were still present although greatly improved

(*) *M* Male, *F* Female

Clinically, OLL lesions are similar to OLP. Reticular, papular, atrophic, erosive/ulcerated, and plaque patterns [29–32] can be observed with or without the presence of skin lesions. OLL is usually unilateral [1, 2]. Since the histopathological aspects of OLL and OLP may be very similar, the clinical information, including the temporal association of the occurrence of the lesions and the use of a drug is important to establish OLL diagnosis related to drugs [2]. However, unlike OLP, the subepithelial inflammatory infiltrate of OLL may contain, beyond lymphocytes, a greater number of eosinophils and/or plasma cells, which may be more diffuse and extend more deeply, or present a perivascular distribution [1].

The classification of these lesions and methodologies for clinical diagnosis varies among the studies. Standardization of diagnostic criteria is a challenge and is extremely important to ensure the validity of studies and the patient’s management and prognosis. Recently, Aguirre-Urizar et al. [32] proposed, in agreement with the World Health Organization [31], that OLP and OLL should be grouped into a term called “oral lichenoid disease”. However, the American Academy of Oral and Maxillofacial Pathology [2] believes that the distinction between these lesions is necessary since they have different biological behaviors. The percentage of malignant transformation of the OLL varies widely in the literature, from 2.1 to 72% [8–10, 33, 34], which indicates a greater malignant potential of OLL compared to OLP (0,4% a 12,5%) [8, 33, 35–41].

## Case presentation

The two patients described in this paper were attended at the Oral Diagnosis Outpatient Clinic of the Antônio Pedro University Hospital, Fluminense Federal University, Niterói - RJ, Brazil, and signed an informed consent form.

### Case 1

A 51-year-old black male patient was referred by the hematology service to the Oral Diagnosis Outpatient Clinic to evaluate a two-month evolution ulcerated oral lesions with symptoms of burning and pain. During anamnesis, the patient reported a history of B-cell NHL diagnosed 12 months before, which was treated with eight cycles of R-CVP chemotherapy (rituximab 700 mg, vincristine 2 mg, cyclophosphamide 1414 mg, and prednisone 60 mg). Concomitantly, the patient was in use of antifungal and antibacterial prophylactic medications (acyclovir 200 mg and a combination of sulphamethoxazole 400 mg and trimethoprim 80 mg). After the third chemotherapy session, the patient observed the presence of anal lesions. After the end of chemotherapy, acyclovir and the combination of sulphamethoxazole and trimethoprim were maintained for more 7 months, at which time the patient observed the presence of ulcerated oral and penile lesions. The patient denied smoking and alcohol consumption, and his laboratory tests were negative for syphilis (Venereal Disease Research Laboratory, VDRL), hepatitis B (Hepatitis B surface antigen, HBsAg), and hepatitis C (antibody to hepatitis C virus, anti-HCV).

**Fig. 1:**
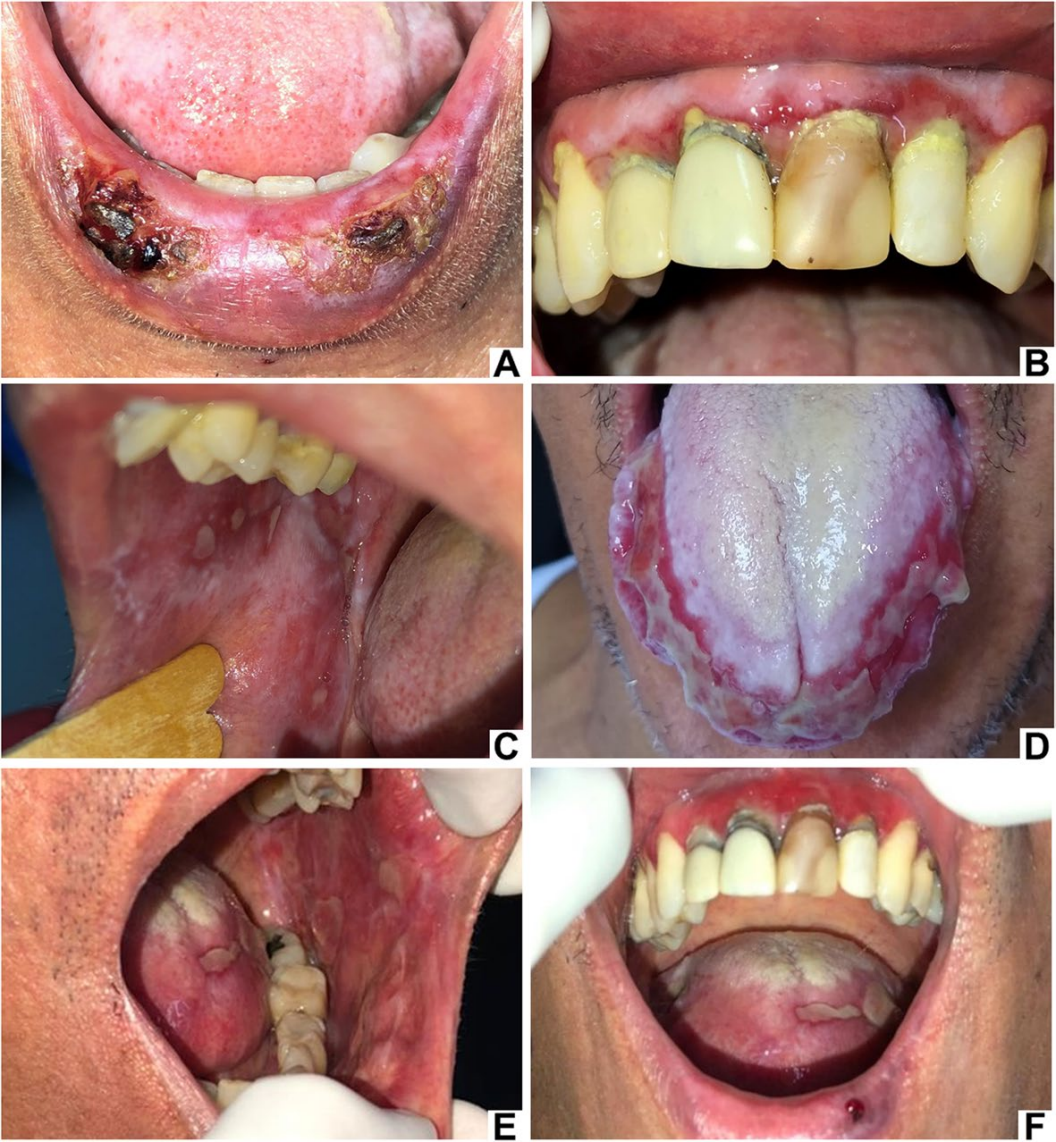
Clinical aspects of case 1 during the follow-up. Lesions with an erosive/ulcer pattern and presence of crusts on the lower lip (**A**). Lesions with plaque pattern on the attached gingiva and atrophic pattern on the marginal gingiva of the upper incisor region - desquamative gingivitis (**B**). Lesions with reticular, plaque, and erosive/ulcerated patterns on the right buccal mucosa (**C**). Erosive/ulcerated pattern on the right and left lateral border and tip of the tongue. A white-coated tongue was also observed (**D**). Current clinical status of the patient. Erosive/ulcerated pattern on the buccal mucosa and tongue (**E**). Atrophic pattern on the gingiva - desquamative gingivitis and erosive/ulcer pattern on the tongue and lower lip (**F**)

In the first appointment, intraoral examination revealed bilateral lesions on the buccal mucosa with reticular, atrophic, and erosive/ulcerated patterns. Moreover, lesions with atrophic and erosive/ulcerated patterns, and a white plaque were present on the lower lip, as well as a plaque on the upper attached gingiva. During the follow-up, the development of other patterns was observed, with a worsening of the condition and involvement of other anatomical regions (labial and lower gingival mucosa, and the dorsum and lateral border of the tongue) (Fig. 1A, B, C, and D).

**Fig. 2:**
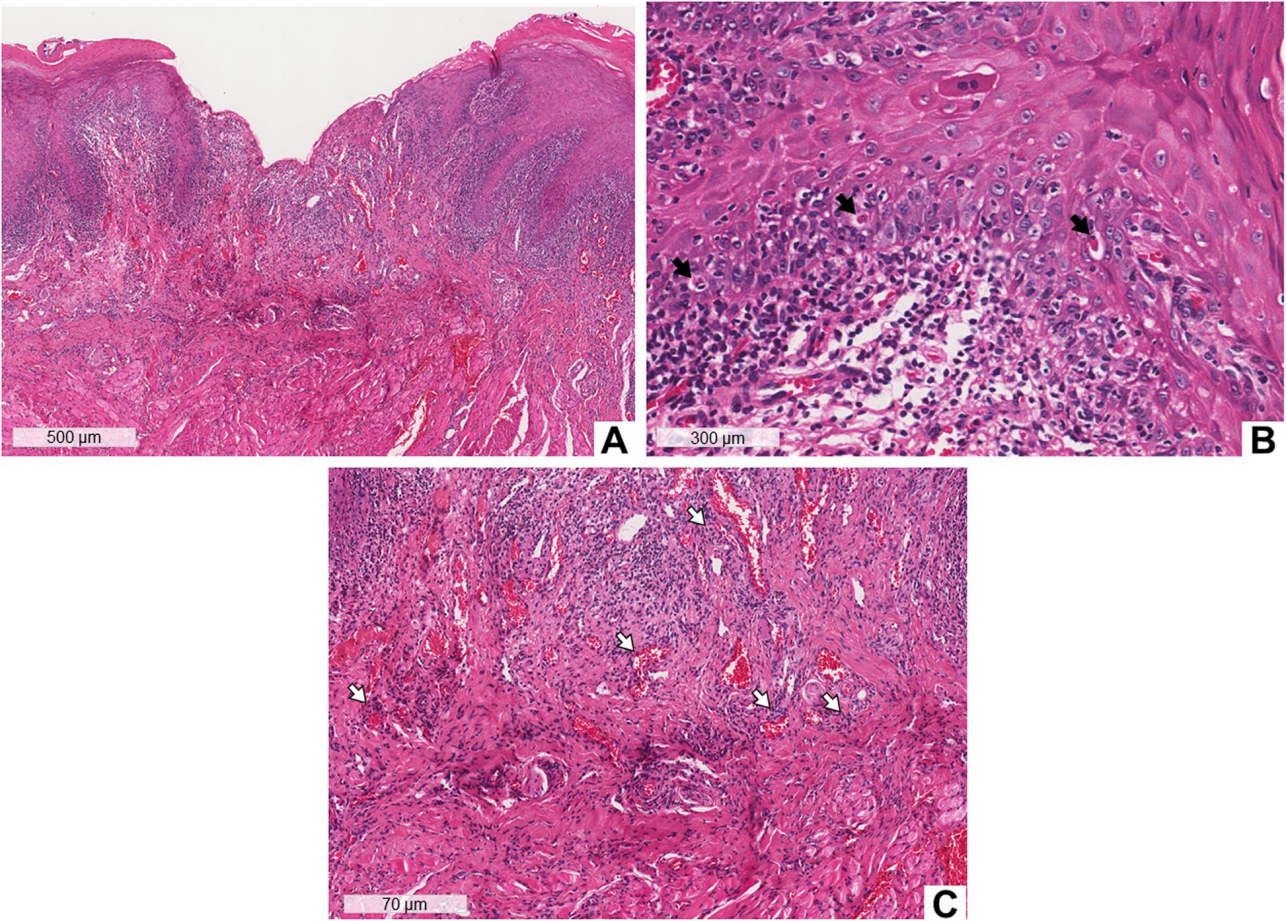
Histopathological aspects of case 1. Biopsy of the lateral border of the tongue. Histological section stained with hematoxylin and eosin (HE), showing mucosa covered by hyperparakeratinized squamous epithelium, with the presence of an ulcer. There is a subepithelial “band” of intense inflammatory infiltrate (**A**). At higher magnification, lymphocyte exocytosis, hydropic degeneration of the basal layer, and Civatte bodies (black arrows) are observed (**B**). Perivascular inflammatory infiltrate (white arrows) (**C**)

An incisional biopsy was performed on the left lateral border of the tongue. The histopathological examination showed a mucosa covered by hyperparakeratinized squamous epithelium, with the presence of ulcer (Fig. 2A), exocytosis of lymphocytes and neutrophils, degeneration of basal cells layer, and Civatte bodies (Fig. 2B). In the connective tissue, there was a subepithelial “band” (Fig. 2A) and a perivascular (Fig. 2C) chronic lymphocytic inflammatory infiltrate. Immunofluorescence analysis was negative for C3 and IgG. The clinical and histopathological aspects were compatible with OLL related to drugs. The diagnosis was confirmed by the temporal association of the onset of oral lesions and the use of chemotherapy drugs and other medications.

The treatment included the use of topical and systemic corticosteroids (clobetasol propionate 0.5 mg/4x daily; prednisone 40 mg daily), as well as topical and systemic antifungals (nystatin 1:100,000 IU/ML/21 days; fluconazole 150 mg once a day/3 days) due to the presence of oral candidiasis confirmed by cytopathological examination. The patient did not come for a follow-up and evaded treatment for 10 months.

Two years and a half after finishing the chemotherapy treatment and 2 years of using acyclovir, sulphamethoxazole and trimethoprim, an improvement in the clinical condition was observed with the use of topical and systemic corticosteroid. However, after the discontinuation of systemic corticosteroid, the lesions exacerbated, despite the use of topical corticosteroids (Fig. 1E and F).

### Case 2

A 32-year-old black male patient was referred by the emergency clinic to the Oral Diagnosis Outpatient Clinic for evaluation of ulcerated oral lesions with symptoms of burning and pain for approximately 7 months. Past medical history included asthma, bronchitis, and the diagnosis of T-cell lymphoblastic NHL one and half year before. The treatment for T-cell lymphoblastic NHL was eight cycles of the Hyper-CVAD chemotherapy protocol (12 mg ondansetron, 300 mg/m^2^ cyclophosphamide, 2 mg vincristine, 5 mg/m^2^ doxorubicin, 40 mg dexamethasone, mesna 600 mg/m^2^, methotrexate 800 mg/m^2^, folinic acid 50 mg, methylprednisolone 50 mg, and cytarabine 3000 mg/m^2^). Also, a prophylactic treatment for infection (acyclovir 200 mg, a combination of sulfamethoxazole 800 mg with trimethoprim 160 mg and a combination of amoxicillin 500 mg with clavulanate potassium 125 mg) was performed. Later, he started a POMP maintenance regimen (vincristine 2 mg, methotrexate 20 mg/m^2^, mercaptopurine 50 mg, and prednisone 200 mg), remaining on acyclovir and a combination of sulphamethoxazole and trimethoprim. In the last cycle of the Hyper-CVAD chemotherapy treatment, he observed the presence of ulcerated oral lesions and, later on papules and pustules in the upper trunk area. The patient reported the use in the past of alcohol, tobacco and cocaine, and his laboratory tests were negative for VDRL, HBsAg, and anti-HCV.

**Fig. 3:**
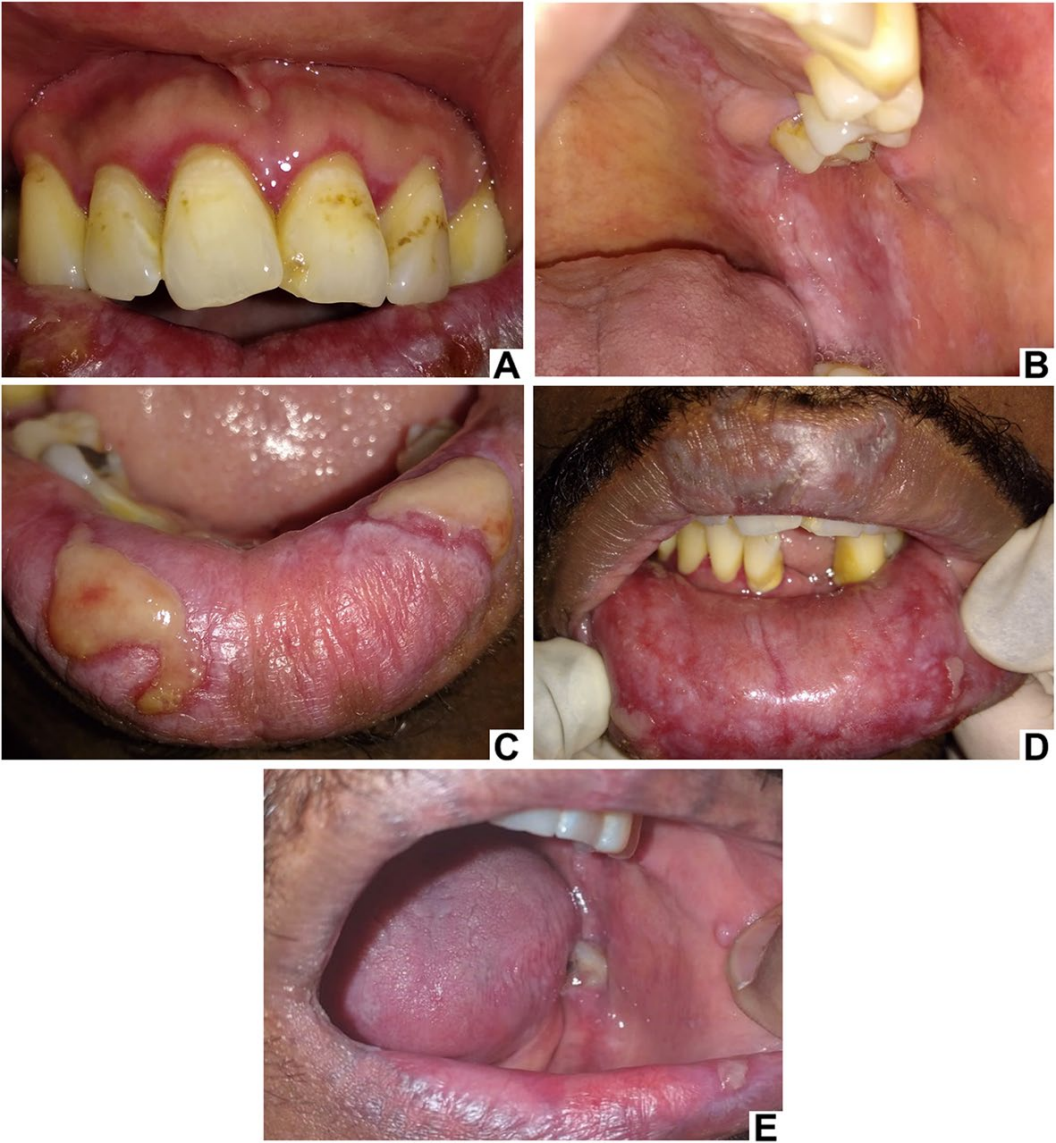
Clinical aspects of case 2. Lesions with atrophic pattern in the gingiva - desquamative gingivitis (**A**). Lesions with atrophic, erosive/ulcerated patterns and plaque on the buccal mucosa and hard and soft palate (**B**). Lesions with erosive/ulcerated pattern on the lower lip (**C**). Reticular pattern on the upper lip and atrophic, erosive/ulcerated patterns, and plaque on the lower labial mucosa (**D**). Remission of lesions on the buccal mucosa, labial mucosa and retromolar area on the left side is noted. Improvement in the clinical aspect and a refractory ulcer on the lower lip after treatment - photo sent by the patient (**E**)

Intraoral examination revealed lesions with reticular patterns in the vermilion of the upper lip; plaque, atrophic, and erosive/ulcerated lesions in the vermillion lower lip and lower labial mucosa; reticular, atrophic, and erosive/ulcerated lesions bilaterally in the buccal mucosa; atrophic and plaque lesions in the retromolar area, soft and hard palate; and atrophic lesions in the upper and lower gingiva (Fig. 3 A, B, C, and D).

**Fig. 4:**
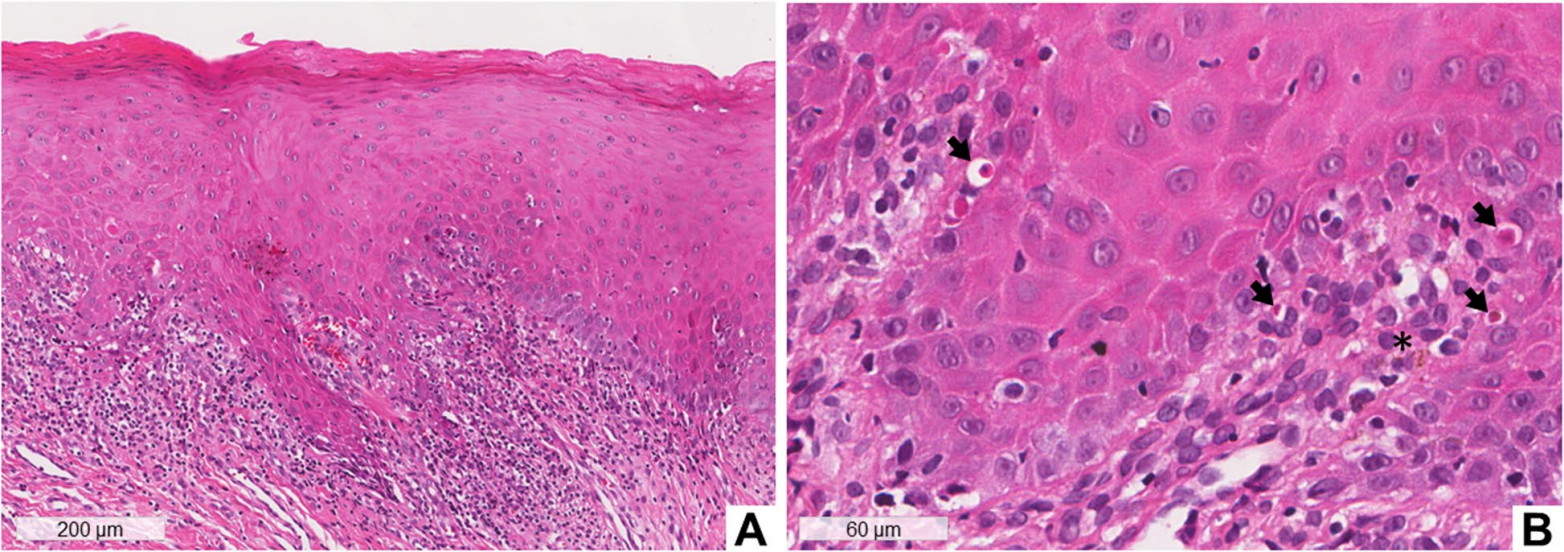
Histopathological aspects of case 2 – Biopsy of the buccal mucosa. Histological section stained with hematoxylin and eosin (HE), showing mucosa covered by hyperparakeratinized squamous epithelium. There is an intense subepithelial “band” of inflammatory infiltrate (**A**). At higher magnification, lymphocyte exocytosis, hydropic degeneration of the basal layer, Civatte bodies (black arrows), and pigmentary incontinence (asterisk) are observed (**B**)

Incisional biopsies were performed on the buccal mucosa and lateral border of the left side of the tongue. The histopathological examination of both biopsies showed a mucosa covered by hyperorthokeratinized and hyperparakeratinized squamous epithelium (Fig. 4A) with areas of acanthosis, lymphocyte exocytosis, degeneration of basal cells layer, and the presence of Civatte bodies (Fig. 4B). In the connective tissue, a subepithelial band-like of a chronic inflammatory infiltrate of lymphocytes (Fig. 4A) and pigmentary incontinence (Fig. 4B) were observed. Immunofluorescence analysis was negative for C3 and IgG. The clinical and histological aspects were compatible with OLL related to drugs, and the diagnosis was confirmed by the temporal association of the use of the chemotherapeutic and prophylactic protocol with the onset of lesions.

The oral lesions treatment included topical and systemic corticosteroids (clobetasol propionate 0.5 mg/4x daily; prednisone 40 mg daily). After 2 years of chemotherapy and 1 year of using acyclovir and the combination of sulphamethoxazole with trimethoprim, there was a significant clinical improvement of the oral lesions, however an ulcer in the lower lip and a white plaque in the gingiva of the upper central incisors region were still present (Fig. 3E).

## Discussion and conclusions

In 2014, Kuten-Shorrer [20] described, for the first time, a case of OLL after the use of high doses of rituximab (750 mg/m^2^) for the treatment of follicular lymphoma. The 43-year-old female patient developed the disease 3 months after the fourth dose and was treated with topical, systemic, and intralesional corticosteroids [20]. The lesions completely resolved within 1 year of treatment, and no new lesions were observed in 6 years of follow-up [20]. In 2019, Giudice et al. [14] reported another case of OLL in a 40-year-old female after treatment of B-cell NHL in the parotid gland with rituximab. After the fifth dose, the patient had ulcerated oral lesions and papular skin lesions [14]. The diagnosis was confirmed by histopathological examination, which showed a “band” of inflammatory infiltrate of lymphocytes near the basement membrane and several areas of keratinocyte necrosis [14]. However, 9 months after rituximab withdrawal, the patient still had oral ulcers [14].

Recently, a case of OLL was also described in a 65-year-old female patient after therapy with pembrolizumab to treat bladder carcinoma [42]. After the fourteenth drug cycle, the patient had large oral ulcers surrounded by irradiated white striae. In this case, the therapy was discontinued and an improvement in symptoms was reported after the introduction of corticosteroid treatment. However, the lesions also did not fully regress [42].

The most reliable means for the diagnosis of OLL related to drugs is to observe the resolution of the reaction after withdrawal of the putative drug and to check whether they return after its reintroduction [43]. Bronny and Thies [18], reported a case of a 59-year-old patient with OLL using several medications for the treatment of multiple diseases. When only sulfamethoxazole, a medication used by both patients in the present study, was suspended, an improvement in the lesions was observed. After the reintroduction of the drug, the lesions returned larger and the symptoms worsened [18]. Usually, the removal of some drugs may be impossible, as they are extremely important for the treatment of NHL. In addition, lesions remission after their discontinuation can take weeks to years [1, 2]. This fact was observed in previous reports [14, 42], as well as in the present cases, in which the oral lesions did not completely resolve after months of discontinuation of the medications.

Another case of OLL refractory to corticosteroid treatment was attributed to bendamustine for the treatment of follicular lymphoma, however the patient was using rituximab at the same time [21]. Therefore, in addition to the challenges mentioned for the diagnosis of OLL, the use of combined drugs in chemotherapy treatment makes difficult the identification of the drugs or drug responsible for the disease, as occurred in the present cases. Besides that, the systemic condition of these patients makes difficult the management of them, such as the use of high doses of corticosteroids and or immunotherapies, even with medical supervision.

Lichenoid lesions and OLP have also been associated with hematological malignancies as a possible anti-tumor immune reaction without the association with the use of chemotherapeutics [44, 45]. It is believed that neoplasms stimulate a cell-mediated immune response against tumor antigens, which cause the generation of self-reactive T cells and cross-react against antigens expressed on epithelial cells [46]. Ouedraogo et al. [27] suggested in a report of four cases of erosive OLL, that the lesions could be an initial sign of paraneoplastic pemphigus associated with hematologic malignancies. Paraneoplastic pemphigus is a rare mucocutaneous autoimmune disease associated with malignancies, especially lymphoproliferative neoplasms, presenting a wide clinical and histopathological features [47]. Among the manifestations, mucocutaneous lichenoid eruptions and a lichenoid histopathological pattern stand out [48].

Cummins et al. [49], in 2007, described four cases of patients with NHL and chronic lymphocytic leukemia who presented paraneoplastic pemphigus with a lichenoid pattern, with no detectable antibodies. Although the diagnosis was paraneoplastic pemphigus, they reported that in three of these patients, the lesions appeared after the use of some chemotherapy drug or combinations of drugs also used in the present cases [49]. Furthermore, we performed immunofluorescence analysis to rule out paraneoplastic pemphigus, and they were negative for C3 and IgG.

The lack of follow-up of patients with OLL related to drugs for a long time, after the resolution of the clinical picture, was noticed in many studies. In the cases reported in this paper, the diagnosis of OLL related to drugs was initially established by the association of histopathological examination and the history of the appearance of lesions after the use of medications. However, we found that, in more than 2 years of follow-up, patients still had dynamic oral lesions after medication withdrawal, with periods of exacerbation, similar to the course of OLP, which makes the differential diagnosis even more challenging.

In conclusion, these two reported cases reinforce the difficulty in differentiating lesions with a lichenoid pattern, due to their clinical and histopathological similarities. Although a well-detailed anamnesis associated with the drug history, temporal relationship of the onset of the lesions, and follow-up of patients are very important for the diagnosis of OLL related to drugs, and its differentiation from OLP. It is noteworthy that these lesions demonstrate a biological behavior that is recalcitrant to corticosteroid treatment, even after withdrawal of the possible inducing medications, which makes the diagnosis and management of these patients even more difficult. Monitoring the behavior and progression of the disease is necessary, not only for treatment but also to validate the initial diagnosis, as the presented two cases demonstrated.

## Data Availability

Not applicable.

## Abbreviations

OLP: Oral lichen planus
OLL: Oral lichenoid lesion
NHL: Non-Hodgkin’s lymphoma
VDRL: Venereal Disease Research Laboratory
HbsAg: Hepatitis B surface antigen
anti-HCV: Antibody to hepatitis C virus
